# Three-Dimensional Observations of a Mesoscale Eddy in the Kuroshio Extension Based on Multiple Platforms

**DOI:** 10.1038/s41597-025-06267-z

**Published:** 2025-12-16

**Authors:** Tianshi Du, Yueqi Zhang, Wu Su, Zhao Jing, Zhaohui Chen, Yumou Qiu, Honghai Zhang, Shaoqiong Yang, Wei Ma

**Affiliations:** 1 Laoshan Laboratory, Qingdao, 266237 China; 2 Frontier Science Center for Deep Ocean Multispheres and Earth System (FDOMES) and Key Laboratory of Physical Oceanography, Ocean University of China, Qingdao, 266100 China; 3 Center for Big Data Research, Peking University, Beijing, 100191 China; 4 School of Mathematical Sciences, Peking University, Beijing, 100191 China; 5 FDOMES, Key Laboratory of Marine Chemistry Theory and Technology, Ministry of Education, and College of Chemistry and Chemical Engineering, Ocean University of China, Qingdao, 266100 China; 6 Key Laboratory of Mechanism Theory and Equipment Design of Ministry of Education, School of Mechanical Engineering, Tianjin University, Tianjin, 300350 China

**Keywords:** Physical oceanography, Ocean sciences, Environmental impact

## Abstract

Mesoscale eddies are the most striking feature in the global upper ocean with notable effects on the climate and marine ecosystem. However, acquiring three-dimensional structure of mesoscale eddies remains challenging as their scale is still beyond the resolution capacity of the current generation of global operational ocean observation system. In this study, we conducted an eddy-oriented survey targeting a cyclonic eddy in the Kuroshio Extension during September 2024. Compared to traditional ship-based observations, our survey is synchronized with the newly launched Surface Water and Ocean Topography (SWOT) mission, and complements shipboard measurements with those from 7 autonomous underwater gliders and 20 surface drifters, capturing the eddy’s three-dimensional structure with spatially high-resolution and wide coverage. The resulting dataset holds significant value for facilitating the calibration and scientific utilization of the SWOT mission, estimating the eddy transport, and validating eddy-resolving ocean forecasting systems.

## Background & Summary

Mesoscale eddies are swirling currents in the ocean^1^ that dominate ocean kinetic energy^2^, play a key role in heat and material transport^3,4^, and strongly interact with the overlaying atmosphere^5,6^. Their nonlinear nature limits analytical descriptions, while numerical models often exhibit substantial biases in simulating eddy statistics^7^, highlighting the need for observational advances. Significant progress was achieved in the 1980s with the advent of surface drifters and satellite altimeters^8,9^, yet these tools are largely restricted to surface measurements, hindering our understanding of the three-dimensional structure of mesoscale eddies.

Since the beginning of the 21^st^ century, the Array for Real-time Geostrophic Oceanography (ARGO) program has greatly advanced the observational capacity of the ocean interior by supplying extensive global profiles of temperature and salinity within 0–2000 m^10^. Nevertheless, acquiring the three-dimensional structure of mesoscale eddies remains challenging because Argo floats, designed for operational oceanography, have a nominal horizontal resolution of ~300 km and a temporal resolution of 10 days that are too sparse to resolve mesoscale eddies^10,11^. To address this limitation, 17 Argo floats with enhanced daily sampling capabilities were deployed in the southeast part of an anticyclonic eddy (AE) during an eddy-oriented survey in the Kuroshio Extension^12^. However, as Argo floats passively drift with ocean currents, it is difficult for them to sample the entire AE.

Ship surveys provide an alternative way to observe mesoscale eddies, which can date back to the early 1970s. The 1970 Soviet Polygon and the 1973 American-British Mid-Ocean Dynamics Experiment revealed the existence of mesoscale eddies in the ocean and offered the first insights into their complexity^13,14^. Eddy-oriented ship surveys in the Kuroshio Extension and the South China Sea uncovered evident regulation of near-inertial internal waves and submesoscale processes by mesoscale eddies^15,16^. However, ship surveys are only capable of sampling a few sections across mesoscale eddies due to their limited navigation time, making it impossible to observe the three-dimensional eddy structure with sufficient spatial coverage.

**Fig. 1:**
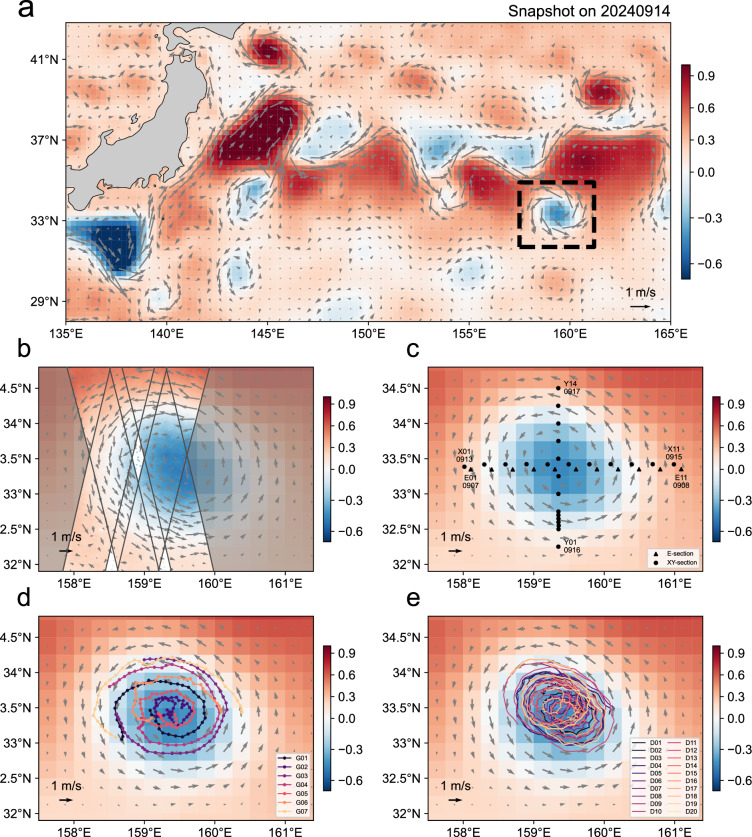
A summary of the eddy-oriented high-resolution observation project in the Kuroshio Extension region during September 7–21, 2024. (**a**) Snapshots of sea level anomaly (SLA, colors) and surface geostrophic current anomaly (vectors) derived from Data Unification and Altimeter Combination System (DUACS) near-real-time products on September 14, 2024. The black dashed line delineates the targeted cyclonic eddy that is shown in (**b**–**e**). (**b**) High-resolution SLA and associated surface geostrophic current anomaly from the Surface Water and Ocean Topography (SWOT) during two overpasses at 02:18 and 13:27 UTC on September 11, 2024. (**c**) Locations of shipboard CTD sites along three sections (E, X, Y). The start and end dates of each section are labeled in MMDD format, (**d**) Trajectories of 7 autonomous underwater gliders during September 13–21. (**e**) Trajectories of 20 surface drifters during September 8–21.

Recently, networked glider arrays have shown strong potential for achieving optimal spatial coverage and adaptive sampling of mesoscale features, providing valuable support for shipboard-only observations^17–20^. The complementary use of drifter and float data has further enhanced the detection and tracking of eddies^21^. Building on these advances, the integration of satellite altimetry with unmanned platforms (e.g., surface drifters, underwater gliders, and Argo floats) has emerged as a powerful strategy for characterizing ocean dynamics^1,21,22^. Motivated by this strategy, we conducted a ship survey on a mesoscale cyclonic eddy (CE) during September 7 to 21, 2024 in the Kuroshio Extension, a well-known hotspot for mesoscale eddies (Fig. 1a). The eddy and the observation period were selected so that the newly launched Surface Water and Ocean Topography (SWOT) mission overpassed the CE-occupied area twice (Fig. 1b). By integrating SLA measurements from SWOT (Fig. 1b), simultaneous hydrographic profiles from shipboard CTD (Conductivity-Temperature-Depth) (Fig. 1c) and autonomous underwater gliders (Fig. 1d), along with surface currents data from surface drifters (Fig. 1e), we constructed a mesoscale eddy dataset with both high resolution and wide coverage in space. A key advancement of our survey is the adaptive glider navigation process, which utilizes real-time current estimates to guide the gliders to move along the pre-designed paths, ensuring full coverage of the targeted CE. In parallel, and in contrast to traditional glider-sampling approaches (e.g., long-term monitoring, fixed transect grids, or virtual moorings), we implemented a time-optimal networking strategy that combined an initial cross-eddy transect with along-flow circumnavigation. This approach enabled the most rapid and efficient reconstruction of the eddy structure to date. As a result, the dataset provides a more complete description on the three-dimensional structure of a mesoscale eddy than the previous ones. In particular, it offers an opportunity to infer vertical velocity from *in situ* data based on the omega equation^23^, supporting further research on the eddy-induced vertical transport, a quantity remaining poorly assessed observationally.

## Methods

Between September 7 and 21, 2024, oceanographic and meteorological data were collected in the Kuroshio Extension region utilizing various *in-situ* platforms. These platforms included shipboard CTD casts (Sea-Bird SBE 911plus), underway Acoustic Doppler Current Profiler (ADCP, TRDI OS-75k), an underway meteorological station (Vaisala AWS430), Petrel-L autonomous underwater gliders (equipped with Seabird Glider Payload CTDs), and SVP surface drifters.

### Shipboard CTD data

During the cruise, three observational sections were conducted to measure temperature and salinity in the upper 800 m via the CTD casts (Fig. 1c). A zonal section (E-section) was first performed during September 7-8, consisting of 11 CTD sites spaced at a 15-nautical-mile interval to provide an initial assessment of the targeted CE. During September 13–17, two additional sections forming a cross-pattern were carried out. The X-section is zonally aligned with 11 CTD sites spaced at a 15-nautical-mile interval. The Y-section is a meridional section with 14 CTD sites. Most CTD sites are spaced at a 15-nautical-mile interval, except for sites Y02 to Y07, which are spaced at a 3-nautical-mile interval to resolve submesoscale processes at the CE edge. Only descending profiles from each CTD cast were retained for further processing.

The CTD data underwent standard manufacturer-recommended quality control (QC) procedures using SBE Data Processing (version 7.26). The steps included data conversion, removal of invalid records, alignment of CTD sensors, cell thermal mass correction, low-pass filtering, loop editing, and bin averaging. These procedures standardized the data into vertical profiles with 1-m vertical bins suitable for subsequent analysis. Measurements within the upper 5 m were excluded to avoid potential contamination from the ship effects.

### Autonomous underwater glider data

To extend the observational coverage beyond the limited area sampled by the research vessel, seven autonomous underwater gliders were deployed. Each glider was programmed to reach a target depth of 1000 meters, with a single profile taking approximately six hours to complete. In total, 277 glider profiles were collected. The horizontal displacement of successive profiles varied depending on glider propulsion and ambient ocean currents, with smaller displacement exhibited near the eddy center where the ocean currents were weaker (Fig. 1d).

The glider data underwent a rigorous QC process provided by the manufacturer. This includes thermal lag correction, compensation for glider-induced motion, and removal of spurious spikes or dropouts^24–26^. Nevertheless, some large deviations remained, indicating that the manufacturer’s built-in QC alone was not sufficient to eliminate all anomalies. To address this, an automated spike detection algorithm based on a moving-window standard deviation was applied to further improve data consistency and reliability. The post-QC data were not subjected to any averaging vertically, preserving the higher resolution for users’ further analysis. It is worth noting that the salinity sensor on glider G06 broke down during the observation period, therefore, this glider did not record salinity measurements.

### Surface drifter data

A total of 20 surface drifters were uniformly deployed when the research vessel moved along the E-section, ensuring representative sampling of surface currents within the CE. These drifters move passively with the currents at a depth of 15 m, with positions recorded every 10 minutes. To ensure data quality, a Median Absolute Deviation (MAD) filter was applied to the distances between consecutive drifter positions. This procedure effectively excluded unrealistic trajectory jumps while preserving the integrity of the physical signal.

### Shipboard underway observations

Throughout the cruise, continuous measurements were collected by a vessel-mounted ADCP and an automated meteorological station.

The research vessel was equipped with a 75 kHz ADCP, which achieved an average profiling depth of approximately 8 m. The raw current velocity data were processed using WinADCP (Version 1.14) for data validation and navigation correction. To further ensure quality, vertical outliers were flagged with a sliding-median and MAD filter, effectively removing unrealistic spikes while preserving the original measurements.

The underway meteorological station was mounted on the forward mast at an average height of 18.5 m above the waterline. All variables, except wind speed and direction, were recorded as 1-min averages, while wind observations were sampled every 3 seconds and subsequently averaged to 1 min for consistency. Data quality was further ensured using a sliding-median and MAD filter to flag and remove outliers, producing a reliable dataset for analysis.

### Adaptive glider navigation

Navigating gliders directly across the CE is infeasible due to their low propulsion speed (~0.3 m s^−1^) compared to the current speed (up to 1 m s^−1^) of the CE. Instead, the paths of individual gliders are designed as concentric circles centered on the CE center to leverage the strong tangential speed of the CE. Although such a glider path design is conceptually simple, several challenging issues need to be addressed during its execution.

**Fig. 2:**
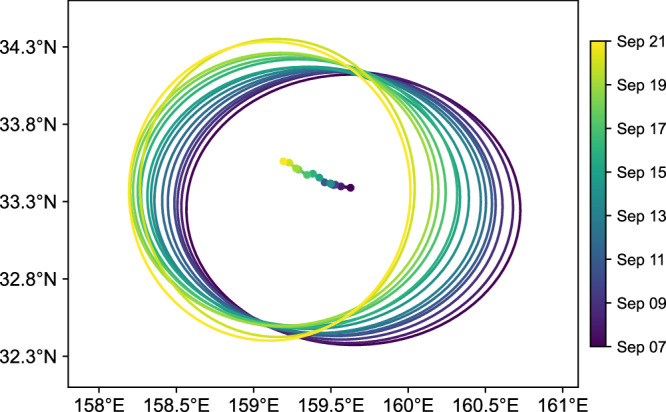
Evolution of the targeted cyclonic eddy during the observation period. Colored dots indicate the eddy center and colored lines delineate the eddy edge computed by fitting an ellipse to the zero contour of SLA.

The first issue is the estimation of the eddy center. The eddy center is determined as the location with minimum surface geostrophic velocity. Two surface geostrophic velocity fields were used, one derived from the gridded SLA provided by the Data Unification and Altimeter Combination System (DUACS) and the other estimated from daily-averaged velocity obtained from the surface drifters. These two surface geostrophic velocity fields are not entirely consistent with each other and have their own advantages and disadvantages. Therefore, the location of the eddy center is determined as the average value derived from both sources. The eddy center was updated on a daily basis. During the survey from September 7–21, the eddy center translated towards northwest at a speed of approximately 3.0 km per day (Fig. 2).

The second issue is the navigation of the gliders to make them move along the targeted circular trajectories in the presence of strong ocean currents. To achieve this, a dynamical glider control scheme was employed^27^. Rather than prescribing waypoints in advance, waypoints were dynamically adjusted every time the glider surfaced based on real-time estimates of ocean currents. This approach accounts for the movement of glider caused by ocean currents, ensuring that its ground velocity is directed toward the targeted trajectories. The surface ocean current field is estimated by fusing the velocities derived from the DUACS data and surface drifter using a 3D-Var assimilation scheme. The subsurface velocity field is regressed from the surface velocity field with the physically-informed regression relationship derived from the GLORYS12 reanalysis dataset^28^.

**Fig. 3:**
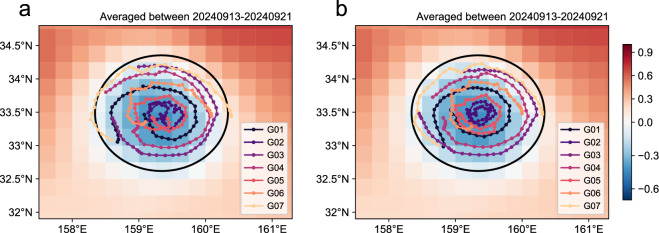
Trajectories of autonomous underwater gliders. (**a**) Absolute trajectories of the gliders. (**b**) Trajectories adjusted relative to the eddy center. The dots mark the positions where the gliders surfaced. Background shading indicates the time-mean sea level anomaly (SLA) derived from the Data Unification and Altimeter Combination System (DUACS) near-real-time products during September 13–21. The back solid line delineates the eddy edge computed by fitting an ellipse to the zero contour of SLA.

It should be noted that the trajectories of gliders are not perfectly circular when referenced to the fixed geographic coordinate system due to the translated eddy center. As the glider profiles are not collected simultaneously but span about two weeks, it is more meaningful to examine their spatial distribution in a coordinate system referenced to the eddy center, thereby minimizing spatial aliasing due to temporal variability. Figure 3a,b compare the absolute glider trajectories with those adjusted relative to the eddy center. Owning to the Adaptive glider navigation strategy, the glider trajectories are distributed slightly more uniformly in the latter coordinate system.

## Data Records

The dataset can be accessed via Zenodo^29^ 10.5281/zenodo.17206966. It is organized into three subfolders according to the observational platforms:

### Shipboard CTD data

This subfolder contains 1-m bin-averaged CTD profile data. The data are archived according to the observational sections, with filenames formatted as: “*CTD_{section_name}_profiles.nc*”. The parameters are listed in Table 1.

**Table 1:** Parameters included in shipboard CTD data file.

Parameter	Header	Unit
time	Time of CTD profile (UTC)	seconds since 2024-01-01 00:00:00
lon	Longitude	degrees East
lat	Latitude	degrees North
pressure	Seawater pressure	dbar
temperature	Seawater temperature	degrees Celsius
salinity	Seawater practical salinity	1
temperature_qc	Quality flag for seawater temperature	/
salinity_qc	Quality flag for seawater salinity	/

### Autonomous underwater glider data

This subfolder contains hydrographic profiles collected by autonomous underwater gliders equipped with CTD sensors. The data are organized into subfolders based on individual glider IDs (G01-G07). Within each subfolder, the profiles are stored as separate files, named according to: “*GLIDER_{glider_ID}_Profile_{profile_number}.nc*”. The parameters are listed in Table 2.

**Table 2:** Parameters included in autonomous underwater glider data file.

Parameter	Header	Unit
ascending_time	Time during ascending phase (UTC)	seconds since 2024-01-01 00:00:00
ascending_pressure	Seawater pressure during ascending phase	dbar
ascending_temperature	Seawater temperature during ascending phase	degrees Celsius
ascending_temperature_qc	QC flag for ascending temperature	/
ascending_salinity	Seawater practical salinity during ascending phase	1
ascending_salinity_qc	QC flag for ascending salinity	/
descending_time	Time during descending phase (UTC)	seconds since 2024-01-01 00:00:00
descending_pressure	Seawater pressure during descending phase	dbar
descending_temperature	Seawater temperature during descending phase	degrees Celsius
descending_temperature_qc	QC flag for descending temperature	/
descending_salinity	Seawater practical salinity during descending phase	1
descending_salinity_qc	QC flag for descending salinity	/

### Surface drifter data

This subfolder includes observations recorded by surface drifters. Each file corresponds to a specific drifter, identified by its unique ID ranging from D01 to D20. The filenames follow the format: “*DRIFTER_{drifter_ID}.nc*”. The parameters are provided in Table 3.

**Table 3:** Parameters included in surface drifter data file.

Parameter	Header	Unit
time	Time of drifters (UTC)	seconds since 2024-01-01 00:00:00
lon	Longitude	degrees East
lat	Latitude	degrees North
temperature	Seawater temperature at surface	degrees Celsius
qc_flag	Quality control flay for drifter data	/

### Underway ADCP data

This subfolder contains measurements collected by the shipboard underway ADCP. The data are organized by observational sections, with filenames following the format: “*ADCP_{section_name}_profiles.nc*”. The parameters are summarized in Table 4.

**Table 4:** Parameters included in underway ADCP data file.

Parameter	Header	Unit
time	Time of ADCP profile (UTC)	seconds since 2024-01-01 00:00:00
lon	Longitude along section	degrees East
lat	Latitude along section	degrees North
depth	Depth below surface	meters
u	Zonal velocity component	m/s
v	Meridional velocity component	m/s
u_qc	Quality flag for zonal velocity	/
v_qc	Quality flag for meridional velocity	/

### Underway meteorological data

This subfolder includes observations recorded by shipboard underway meteorological station. Files are organized by observational section and named using the format: “*Meteorology_{section_name}_profiles.nc*”. A summary of the measured parameters is provided in Table 5.

**Table 5:** Parameters included in underway meteorological data file.

Parameter	Header	Unit
time	Time of observation (UTC)	seconds since 2024-01-01 00:00:00
lon	Longitude along section	degrees East
lat	Latitude along section	degrees North
air_temperature	Air temperature	degrees Celsius
air_pressure	Air pressure	hPa
relative_humidity	Relative humidity	%
u_wind	Zonal wind component	m/s
v_wind	Meridional wind component	m/s
air_temperature_qc	Quality flag for air temperature	/
air_pressure_qc	Quality flag for air pressure	/
relative_humidity_qc	Quality flag for relative humidity	/
u_wind_qc	Quality flag for wind speed	/
v_wind_qc	Quality flag for wind direction	/

## Technical Validation

### Equipment validation

The shipboard CTD data were acquired using a Sea-Bird SBE 911plus system installed aboard R/V *Dongfanghong 3*, which is operated by the Ocean University of China. The instrument system undergoes annual metrological verification in compliance with the National Metrological Verification Regulation *JJG763-2019*, administered by the National Center of Ocean Standards and Metrology. Specifically, prior to the research cruise, all primary sensors (conductivity, temperature, and pressure) of the SBE 911plus system underwent comprehensive calibration procedures from June to July 2024. The Petrel-L autonomous underwater gliders developed by Tianjin University, China, were used in this survey^30^. Each glider was equipped with a Sea-Bird Electronics (SBE) designed glider payload CTD (GPCTD, pumped). All sensors onboard the Petrel-L gliders used in this survey were calibrated at the National Center of Ocean Standards and Metrology in July 2024, following the same procedures applied to the shipboard CTD, prior to deployment. Therefore, the equipment used during this cruise was technical sound, providing high-confidence, accurate, and reliable measurements.

### Cross-validation of hydrographic profiles

**Fig. 4:**
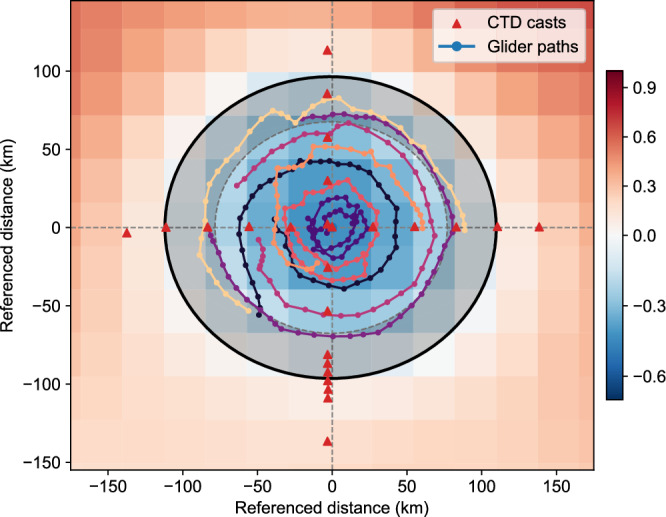
Definition of eddy-core and eddy-edge profiles. Profiles located within the innermost 70% area of the eddy are labeled as eddy-core profiles (non-shaded circle), while those located between the outer edge of the eddy-core area and the eddy edge are labeled as eddy-edge profiles. Here, the eddy edge is computed by fitting an ellipse to the zero contour of SLA.

To further validate the dataset, we conducted a cross-platform comparison based on the shipboard CTD casts and glider observations. In view that hydrographic properties can differ substantially between the eddy core and its edge, profiles are classified into two groups (eddy-core and eddy-edge profiles) according to their relative locations referenced to the eddy center (Fig. 4). In total, 294 profiles were collected within the eddy, of which 216 (206 glider, and 10 CTD) were classified as eddy-core group and 78 (71 glider, and 7 CTD) as eddy-edge group.

**Fig. 5:**
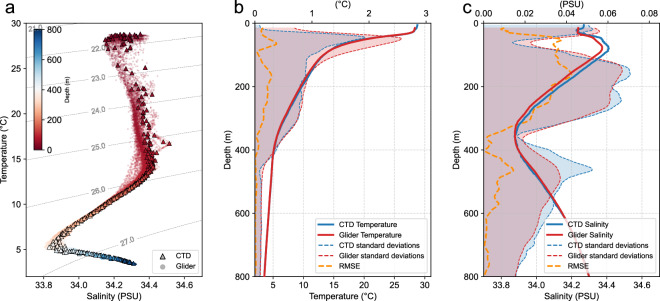
Cross-validation of hydrographic profiles measured by the shipboard CTD casts and gliders in the eddy-core area. (**a**) Temperature-salinity (T-S) diagram derived from the shipboard CTD casts (triangles) and gliders (dots), with color indicating sampling depth. (**b**) Ensemble averages of temperature profiles from shipboard CTD casts (blue) and gliders (red). Shaded areas represent the standard deviations within each ensemble, and their absolute difference is shown in yellow; both are referenced to the top axes. (**c**) Same as (**b**) but for the temperature.

Figure 5 presents the hydrographic properties of eddy-core profiles measured by the two kinds of platforms (i.e., shipboard CTD casts and gliders). The temperature-salinity (T-S) diagram demonstrates a tight T-S relationship below 300 m that is consistently captured by the measurements from the two kinds of platforms (Fig. 5a). The inter-platform consistency is further supported by comparing ensemble mean profiles of temperature and salinity from the two kinds of platforms. Their ensemble mean temperature profiles differ by 0.09 °C throughout the upper 800 m, much smaller than the standard deviations of the profiles within each ensemble (Fig. 5b). So is the case for the salinity profile (Fig. 5c).

**Fig. 6:**
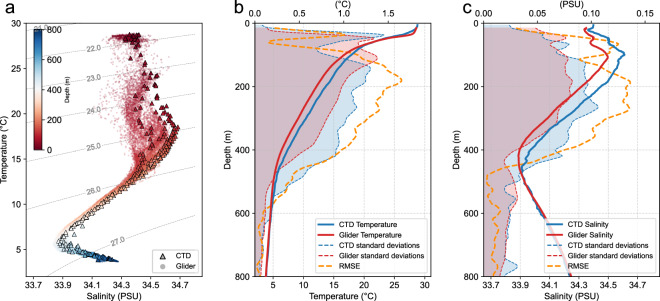
Same as Fig. 5 but for the hydrographic profiles in the eddy-edge area.

**Fig. 7:**
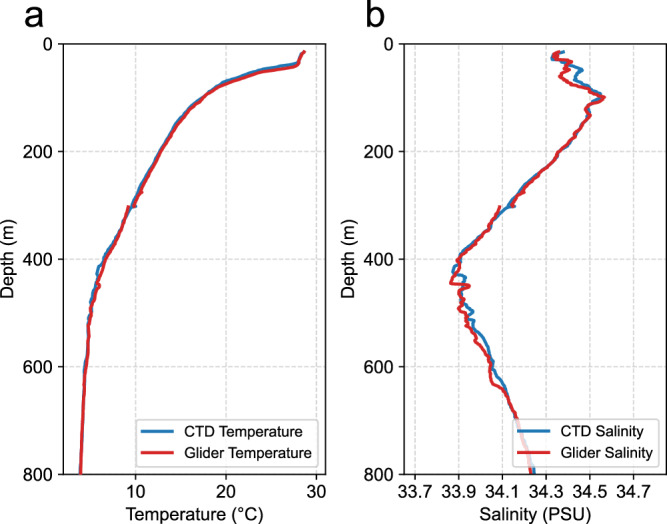
Paired temperature and salinity profiles collected by shipboard CTD casts and gliders in the eddy-edge area. Profiles collected by shipboard CTD casts and gliders are considered as pairs in the spatio-temporal domain if they were located within 10 km of each other and collected within a 6-hour time window. The lines correspond to the ensemble mean of the individual pairs.

The T-S relationship becomes less tight for the eddy-edge profiles, suggesting significant changes in water mass properties around the eddy edge (Fig. 6). The difference of ensemble averages from the two kinds of platforms in the eddy-edge area is larger than that in the eddy-core area and comparable to the within-ensemble standard deviations (Figs. 5, 6). It is important to note that the profiles from the two kinds of platforms do not coincide in the spatio-temporal domain. The larger difference of ensemble averages in the eddy-edge area does not mean the lower observation quality in this region but is probably due to the stronger spatio-temporal variability of the temperature and salinity there. Indeed, for profiles collected in close spatial and temporal proximity, the temperature and salinity profiles from the two kinds of platforms agree well with each other (Fig. 7).

In summary, the high degree of consistency between the measurements from the shipboard CTD casts and gliders provides critical assurance of the quality of the observations, thereby supporting the credibility of subsequent analyses based on these observations.

## Usage Notes

The eddy-oriented survey in the Kuroshio Extension region provides detailed three-dimensional hydrographic observations and surface velocity observations of a CE. On the one hand this dataset offers a robust foundation for validating and refining eddy-resolving ocean forecasts. On the other hand, it has important scientific applications some of which are briefly discussed below.

**Fig. 8:**
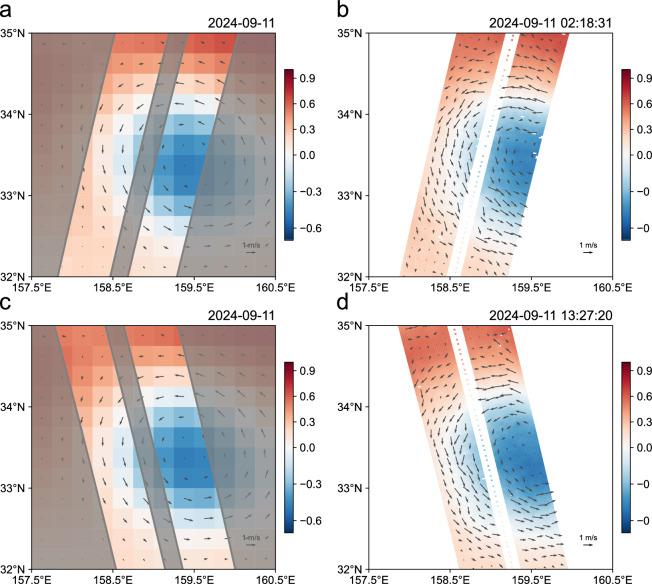
Comparison of SLA from Data Unification and Altimeter Combination System (DUACS) data product and Surface Water and Ocean Topography (SWOT). Sea level anomaly (SLA) and derived geostrophic velocity from the DUACS data product (**a,c**) and SWOT (**b,d**).

A particularly valuable application of this dataset lies in validating and interpreting the SLA measurements from the newly launched SWOT mission^31^, which passed over the CE twice during the observation period. The observed discrepancies between SWOT and the DUACS data product highlight the need for *in situ* validation (Fig. 8). Our high-resolution hydrographic and velocity observations can not only assess the accuracy and noise level of the SWOT-measured SLA, but also aid in decomposing it into balanced and unbalanced oceanic processes^32,33^.

The three-dimensional hydrographic observations and surface velocity observations can be further integrated to estimate the three-dimensional geostrophic velocity of the CE by utilizing the thermal wind formula, enabling the inference of vertical velocity using a quasi-geostrophic version of the omega equation^23^. This provides a rare opportunity to investigate the eddy-induced vertical transport, which remains poorly assessed observationally.

To facilitate further research, the eddy center trajectory from September 1 to 26 has been provided as a supplementary file (“*eddy_center.xlsx*”). This file includes the daily position of the eddy center, allowing users to analyze its spatial evolution and compute the position of observations referenced to the eddy center. In addition, a Python script (“*eddy_movement_adjustment.py*”) is included with the dataset to assist users in computing the position of observations referenced to the eddy center.

## Data Availability

The dataset can be accessed via Zenodo 10.5281/zenodo.17206966.

## Code availability

All custom codes used for data processing and quality control are openly provided within the dataset. Additional scripts that may assist users in further data analysis are also included.

## References

[1] Zhang, Z., Wang, G., Wang, H. & Liu, H. Three-Dimensional Structure of Oceanic Mesoscale Eddies. *Ocean-Land-Atmosphere Res.* **3**, 0051 (2024).

[2] Wunsch, C. & Ferrari, R. Vertical mixing, energy, and the general circulation of the oceans. *Annu. Rev. Fluid Mech.* **36**, 281–314 (2004).

[3] Zhang, Z., Wang, W. & Qiu, B. Oceanic mass transport by mesoscale eddies. *Science* **345**, 322–324 (2014). 25035491 10.1126/science.1252418

[4] Dong, C., McWilliams, J. C., Liu, Y. & Chen, D. Global heat and salt transports by eddy movement. *Nat. Commun.* **5**, 3294 (2014). 24534770 10.1038/ncomms4294

[5] Small, R. J. *et al*. Air–sea interaction over ocean fronts and eddies. *Dyn. Atmospheres Oceans* **45**, 274–319 (2008).

[6] Seo, H. *et al*. Ocean Mesoscale and Frontal-Scale Ocean–Atmosphere Interactions and Influence on Large-Scale Climate: A Review. *J. Clim.* **36**, 1981–2013 (2023).

[7] Griffies, S. M. *et al*. Impacts on ocean heat from transient mesoscale eddies in a hierarchy of climate models. *J. Clim.* **28**, 952–977 (2015).

[8] Richardson, P. L. Caribbean Current and eddies as observed by surface drifters. *Deep Sea Res. Part II Top. Stud. Oceanogr.* **52**, 429–463 (2005).

[9] Chelton, D. B., Schlax, M. G., Samelson, R. M. & de Szoeke, R. A. Global observations of large oceanic eddies. *Geophys. Res. Lett*. **34** (2007).

[10] Johnson, G. C. *et al*. Argo—Two Decades: Global Oceanography, Revolutionized. *Annu. Rev. Mar. Sci.* **14**, 379–403 (2022). 10.1146/annurev-marine-022521-102008 34102064

[11] Sánchez-Román, A., Ruiz, S., Pascual, A., Mourre, B. & Guinehut, S. On the mesoscale monitoring capability of Argo floats in the Mediterranean Sea. *Ocean Sci.* **13**, 223–234 (2017).

[12] Xu, L. *et al*. Observing mesoscale eddy effects on mode-water subduction and transport in the North Pacific. *Nat. Commun.* **7**, 10505 (2016). 26829888 10.1038/ncomms10505 PMC4740428

[13] Hartline, B. K. POLYMODE: Exploring the Undersea Weather. *Science* **205**, 571–573 (1979). 17729670 10.1126/science.205.4406.571

[14] The MODE Group. The Mid-Ocean Dynamics Experiment. *Deep Sea Res.* **25**, 859–910 (1978).

[15] Li, Q. *et al*. Enhanced Near-Inertial Waves and Turbulent Diapycnal Mixing Observed in a Cold- and Warm-Core Eddy in the Kuroshio Extension Region. *J. Phys. Oceanogr.* **52**, 1849–1866 (2022).

[16] Cao, H., Jing, Z. & Fox-Kemper, B. Scale-Dependent Vertical Heat Transport Inferred From Quasi-Synoptic Submesoscale-Resolving Observations. *Geophys. Res. Lett.* **51**, e2024GL110190 (2024).

[17] Tang, H. *et al*. Vigorous Forced Submesoscale Instability Within an Anticyclonic Eddy During Tropical Cyclone “Haitang” From Glider Array Observations. *J. Geophys. Res. Oceans* **130**, e2024JC021396 (2025).

[18] Shang, X. *et al*. Submesoscale Motions Driven by Down-Front Wind Around an Anticyclonic Eddy With a Cold Core. *J. Geophys. Res. Oceans* **128**, e2022JC019173 (2023).

[19] Li, S., Zhang, F., Wang, S., Wang, Y. & Yang, S. Constructing the three-dimensional structure of an anticyclonic eddy with the optimal configuration of an underwater glider network. *Appl. Ocean Res.* **95**, 101893 (2020).

[20] Pelland, N. A., Bennett, J. S., Steinberg, J. M. & Eriksen, C. C. Automated Glider Tracking of a California Undercurrent Eddy Using the Extended Kalman Filter, 10.1175/JTECH-D-18-0126.1 (2018).

[21] Pascual, A. *et al*. A Multiplatform Experiment to Unravel Meso- and Submesoscale Processes in an Intense Front (AlborEx). *Front. Mar. Sci*. **4** (2017).

[22] Farrar, J. T. *et al*. S-MODE: The Sub-Mesoscale Ocean Dynamics Experiment. *Bull. Am. Meteorol. Soc.* **106**, E657–E677 (2025).

[23] Liu, L., Xue, H. & Sasaki, H. Diagnosing Subsurface Vertical Velocities from High-Resolution Sea Surface Fields. *J. Phys. Oceanogr.* **51**, 1353–1373 (2021).

[24] Luo, C. *et al*. Analysis of glider motion effect on the performance of pumped CTD: Implications for vehicle operation and data processing. *Ocean Eng.* **285**, 115383 (2023).

[25] Luo, C. *et al*. Model-based many-objective optimization for control parameters of underwater glider considering long-term high-quality CTD measurements. *Ocean Eng.* **293**, 116591 (2024).

[26] Wang, Y. *et al*. Modified Thermal Lag Correction of CTD Data from Underwater Gliders. *J. Coast. Res.* **99**, 137–143 (2020).

[27] Su, W., E, X., Jing, Z. & Chen, S. X. Glider Path Design and Control for Reconstructing Three-Dimensional Structures of Oceanic Mesoscale Eddies. Preprint at, 10.48550/arXiv.2504.18936 (2025).

[28] Jean-Michel, L. *et al*. The Copernicus Global 1/12° Oceanic and Sea Ice GLORYS12 Reanalysis. *Front. Earth Sci*. **9** (2021).

[29] Du, T. & Zhang, Y. Three-Dimensional Observations of a Mesoscale Eddy in the Kuroshio Extension Based on Multiple Platforms (Version v2) [Data set]. *Zenodo.*10.5281/zenodo.17206966 (2025). 10.1038/s41597-025-06267-z PMC12749307 41402375

[30] Zhang, R. *et al*. Ocean Current-Aided Localization and Navigation for Underwater Gliders With Information Matching Algorithm. *IEEE Sens. J.* **21**, 26104–26114 (2021).

[31] Fu, L.-L. *et al*. The Surface Water and Ocean Topography Mission: A Breakthrough in Radar Remote Sensing of the Ocean and Land Surface Water. *Geophys. Res. Lett.* **51**, e2023GL107652 (2024).

[32] Qiu, B. *et al*. Seasonality in Transition Scale from Balanced to Unbalanced Motions in the World Ocean. *J. Phys. Oceanogr.* **48**, 591–605 (2018).

[33] Archer, M., Wang, J., Klein, P., Dibarboure, G. & Fu, L.-L. Wide-swath satellite altimetry unveils global submesoscale ocean dynamics. *Nature* **640**, 691–696 (2025). 40240853 10.1038/s41586-025-08722-8 PMC12003163

